# Evaluation of Peri-Implantitis Bone Defect Healing: Comparing the Efficacy of Small-Particle Dentin and Bio-Oss in Bone Density Attenuation

**DOI:** 10.3390/jcm13164638

**Published:** 2024-08-08

**Authors:** Michał Łobacz, Katarzyna Wieczorek, Paulina Mertowska, Sebastian Mertowski, Marek Kos, Ewelina Grywalska, Grzegorz Hajduk, Mansur Rahnama-Hezavah

**Affiliations:** 1 Chair and Department of Oral Surgery, Medical University of Lublin, 20-093 Lublin, Polandesso53@wp.pl (G.H.); mansur.rahnama-hezavah@umlub.pl (M.R.-H.); 2 Department of Experimental Immunology, Medical University of Lublin, 20-093 Lublin, Poland; paulinamertowska@umlub.pl (P.M.); sebastian.mertowski@umlub.pl (S.M.); ewelina.grywalska@umlub.pl (E.G.); 3 Department of Public Health, Medical University of Lublin, 20-400 Lublin, Poland; marekkos@umlub.pl

**Keywords:** periimplantitis, bone substitute materials, small-particle dentin, Bio-Oss, bone regeneration

## Abstract

**Introduction:** Peri-implantitis is a serious complication in dental implantology that, if left untreated, may lead to implant loss and systemic diseases. Effective regeneration of bone defects resulting from peri-implantitis is crucial to maintaining the functionality of dental implants. **Purpose of the Study:** The study aimed to compare the effectiveness of fine-particle dentin and Bio-Oss in the reconstruction of bone defects caused by peri-implantitis. **Materials and Methods:** The study included a comprehensive radiological assessment of changes in bone density over time. Bone density was assessed using Hounsfield Units (HUs) as a measure of bone attenuation, with radiological assessments performed at 8- and 12-week intervals during the healing process. The study included participants ranging in age from 30 to 65 years. Fifty-seven patients were divided into three groups: 22 patients received small-particle dentin, 15 received Bio-Oss, and 20 controls without bone substitute material. **Results:** The fine-dentin group showed a 20% increase in bone density after 8 weeks (*p* < 0.05), while the Bio-Oss group showed a 15% increase after 12 weeks (*p* < 0.05). The control group showed minimal changes in bone density (5% after 12 weeks), which was not statistically significant. Clinical evaluations showed 95% successful integration in the fine dentin group, 85% in the Bio-Oss group, and 70% in the control group. The fine-dentin group showed a 20% increase in bone density after 8 weeks (*p* < 0.05), while the Bio-Oss group showed a 15% increase after 12 weeks (*p* < 0.05). The control group showed minimal changes in bone density (5% after 12 weeks), which was not statistically significant. Clinical evaluations showed 95% successful integration in the fine-dentin group, 85% in the Bio-Oss group, and 70% in the control group. **Conclusions**: Both fine-particle dentin and Bio-Oss significantly improved bone density compared to the control group. Fine-particle dentin is suitable for immediate bone regeneration due to its rapid initial regeneration, while Bio-Oss provides long-term support, ideal for maintaining implant stability over a longer period of time. The results highlight the importance of selecting appropriate bone replacement materials depending on the clinical scenario to improve patient outcomes after dental implant placement.

## 1. Introduction

Peri-implantitis is a serious inflammation of the tissues around dental implants that can lead to implant loss and systemic health problems if not treated appropriately. This disease develops as a result of insufficient osseointegration, which means that the implant is not properly anchored in the bone. This is often accompanied by the multiplication of pathogenic bacterial flora, leading to the formation of a biofilm around the implant [1,2,3]. The development of peri-implantitis is associated with significant changes in the composition of the oral microflora, where harmful pathogens begin to dominate over beneficial commensal bacteria, leading to chronic inflammation. The immune response to this condition includes the excessive production of pro-inflammatory cytokines such as IL-1β, TNF-α, and IL-6, which contributes to the destruction of bone tissue and destabilization of the implant. Chronic inflammation can also affect the patient’s overall health, emphasizing the importance of early diagnosis and effective treatment [4,5,6,7]. In clinical practice, the diagnosis of peri-implantitis is based on clinical observations, such as the presence of deep pockets around the implant, bleeding on probing, and possible discharge of purulent fluid. Treatment requires an interdisciplinary approach, including thorough cleaning of implants, the use of antibiotics and sometimes surgical tissue correction [8,9,10].

**Figure 1:**
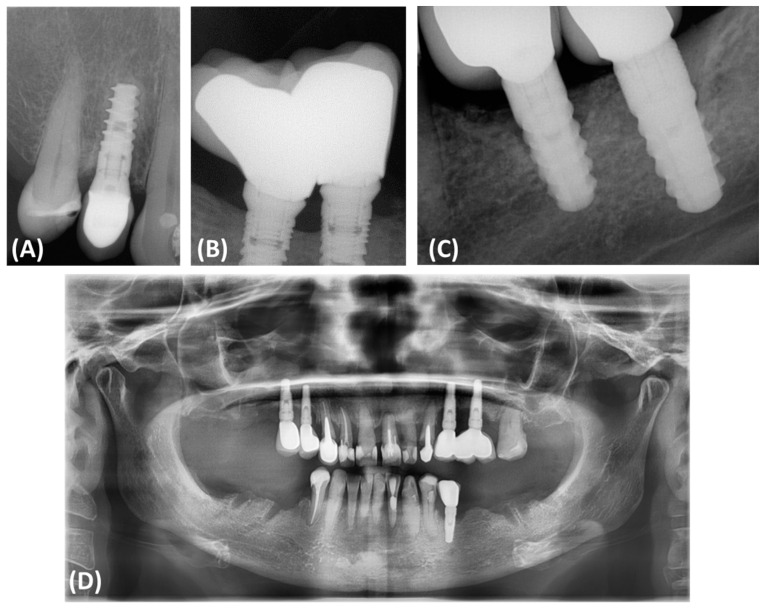
Classification and assessment of the morphology of bone defects associated with peri-implantitis. (**A**) Radiographic description of bone defects associated with peri-implantitis subosseous defect (Class I); (**B**) radiographic description of bone defects associated with peri-implantitis horizontal defect (Class II); (**C**) radiographic description of bone defects associated with peri-implantitis complex defect (Class III); (**D**) panoramic radiograph of a patient illustrating the presence of Class III bone defects around all implanted dental implants.

Peri-implantitis often results in bone defects of various morphology and severity, which directly affects the results of reconstruction and complicates treatment decisions. According to Monje and colleagues, bone defects associated with peri-implantitis often have a subosseous component and include significant buccal bone loss. These defects are classified based on morphology and the extent of bone damage, which has an important impact on treatment decisions (Figure 1). Peripheral defects are usually more amenable to regenerative procedures aimed at rebuilding the bone around the implant. However, open defects, especially those with significant cheekbone loss, pose a greater challenge and often require more complex and less predictable treatment strategies [11,12,13,14,15].

**Figure 2:**
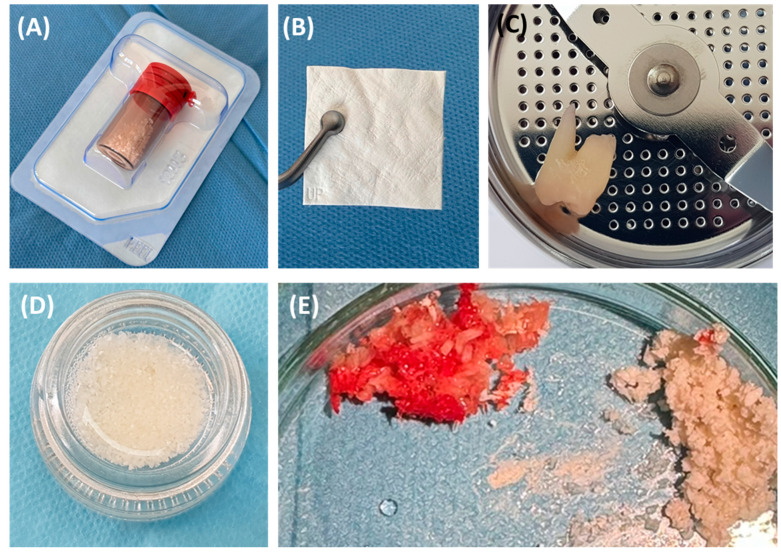
The most commonly used bone substitute materials. (**A**) Xenograft material; (**B**) barrier membrane; (**C**) impacted tooth before processing procedure; (**D**) obtained small-particle dentin; (**E**) comparison of autogenous bone chips and xenograft material.

Effective management strategies must include both the elimination of infection and bone stabilization or regeneration to prevent further deterioration and ensure implant durability. This requires a thorough diagnostic assessment using advanced imaging techniques, such as cone beam computed tomography (CBCT), which allows for precisely determining the morphology of the defect and planning appropriate therapeutic interventions [16,17]. The use of bone substitute materials is crucial in the rehabilitation of dental implants damaged due to peri-implantitis. Such defects often require significant regenerative interventions to ensure the stability and functionality of the implants. According to the consensus of the European Workshop of Periodontology on Bone Regeneration, a variety of bone replacement materials, including xenografts, allografts, and synthetic options, have been used to successfully regenerate alveolar defects [1,18,19]. The use of fine-particle dentin is also common (Figure 2).

The regeneration of peri-implantitis defects is a challenge due to the complex biological conditions in inflamed tissues, the presence of bacterial biofilm, and the complicated nature of bone healing processes. Therefore, a thorough evaluation of available bone replacement materials is necessary. Such an assessment is crucial, especially in the context of peri-implantitis, where bone quality and regenerative conditions are significantly deteriorated by these factors. Comparing the performance of these materials in specific clinical settings allows for more accurate clinical decision-making and improved treatment outcomes based on defect characteristics and the overall clinical scenario.

The study aims to evaluate and compare the effectiveness of fine-particle dentin and Bio-Oss in the reconstruction of bone defects resulting from periimplantitis. The study aims to obtain knowledge about the suitability of these materials for immediate and long-term bone regeneration.

## 2. Materials and Methods

### 2.1. Patient Characteristics

The study included 57 patients with diagnosed peri-implantitis requiring dental implant extraction. All patients recruited for this study had the same stage of peri-implantitis. The study included adult patients aged between 18 and 60 years who did not have any systemic problems, such as diabetes, autoimmune diseases, heart diseases, kidney, or liver diseases. An important criterion was also the lack of medications that may affect the healing process, such as steroids, immunosuppressants, or anticancer drugs. The local condition of the patients included the presence of bone defects around the implants caused by inflammation of the peri-implant tissues. All patients also gave informed consent to participate in the study and perform all study-related procedures. Additionally, the study assessed whether patients had osteoporosis or osteopenia, whether they were treated with antiresorptive drugs (treatment for osteoporosis), and whether they had medication-induced osteonecrosis of the jaw. Patients with any of these conditions were excluded from the study. Patient exclusion criteria included those under 18 years of age, those with any systemic diseases, and those taking medications that may affect the healing process. Pregnant or breastfeeding women, individuals with other inflammatory conditions or pathologies in the oral cavity that may affect the study results, and those who did not give informed consent to participate or undergo related procedures were also excluded. Additionally, patients with a history of treatment at the study sites that could influence the results, such as previous regenerative treatments, were not included in the study. Patients included in the study had a BMI between 18 and 24, with values outside this range constituting an exclusion factor. All patients had missing teeth in the molar regions of the maxilla and mandible. Importantly, none of the patients had previously undergone bone augmentation procedures. All participants were experiencing severe peri-implantitis, which required the explantation of implants and the application of guided bone regeneration techniques. Additionally, the assignment to Group 1 was based on the presence of an asymptomatic wisdom tooth, which provided the material for obtaining small-particle dentin. As a result of the adopted inclusion and exclusion criteria, 39 women (68.42%) and 18 men (31.58%) were included in the study and assigned to three groups based on the appropriate choice of material for clinical application: Small-Particle Dentin Group (22 people), Bio-Oss Group (15 people), and Control Group (20 people). Detailed patient characteristics are presented in Table 1.

Due to the differences in anatomical location observed in individual groups, as well as gender dependencies, detailed data are presented in Table 2 and Table 3.

### 2.2. Characteristics of the Materials Used

#### 2.2.1. Fine-Particle Dentin

Fine-particle dentin, also known as small-particle dentin, is an advanced material used in bone regeneration procedures, especially in dental implantology. It is characterized by very small particles with a diameter of less than 1 mm, which allows for better adhesion and integration with natural bone tissue. Its microporous structure supports the processes of osteoconduction (conduction of bone growth) and osteoinduction (stimulation of new bone formation), and also ensures adequate fluid circulation and enables the penetration of bone-forming cells. Fine-molecular dentin is biocompatible, which means it does not cause immunological or inflammatory reactions, which minimizes the risk of rejection of the material and ensures good tolerance by the patient’s body. This material may come from various sources, including animals (most often cattle), or be synthetic. In the case of materials of animal origin, it is properly cleaned and sterilized to ensure safe use. Fine-molecular dentin is strong enough to provide structural stability at the site of the bone defect while being flexible enough to adapt to the irregularities and shape of the defect. It is gradually reabsorbed by the body and replaced by natural bone tissue, which usually takes several months, depending on the individual characteristics of the patient and the place of application. Thanks to its fine-particle form, fine-particle dentin is easy to apply to the defect site and can be mixed with the patient’s blood or other bioactive substances, which additionally supports the healing process. Numerous clinical studies have shown that this material is effective in bone regeneration, especially in the cases of bone defects caused by inflammation of the tissues around the implant, and safe in long-term use [20,21,22,23,24].

**Figure 3:**
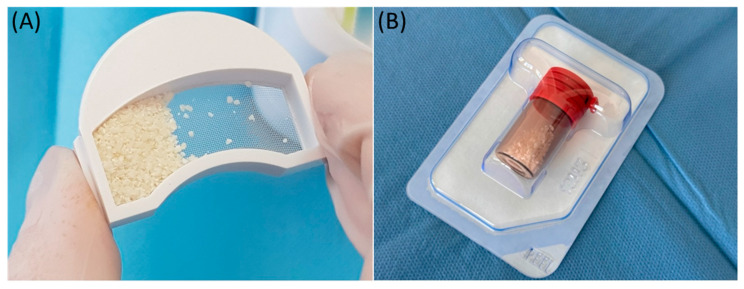
Illustrative photo of the materials used. (**A**) Obtained bone substitute material from the tissues of the processed tooth; (**B**) material of xenogeneic origin—BioOss.

In this study, small-particle dentin from a retained lower wisdom tooth was used. The dentin was prepared according to the protocol provided by Kometabio from the USA. The quantity obtained from grinding one retained lower wisdom tooth was recorded and used for application in the post-extraction socket (Figure 3A).

#### 2.2.2. Bio-Oss

Bio-Oss, a natural bone substitute of bovine origin, is widely used in dentistry and orthopedics due to its unique osteoconductive properties and structural similarity to human bone. It plays a key role in the treatment of bone defects resulting from the inflammation of the peri-implant tissue. It is made from beef bone, which goes through rigorous cleaning and sterilization processes to remove all organic ingredients. This makes this material safe and highly biocompatible. The resulting granular material mimics the mineral composition and porosity of human bone, which facilitates its integration with existing bone tissue and acts as an effective scaffold for new bone growth. This material is mainly used to fill bone defects and strengthen bones in areas affected by peri-implantitis. The use of Bio-Oss significantly improves the structural stability of dental implants, providing a solid base for new bone growth and facilitating the long-term success of the implants. Bio-Oss offers several benefits, including a proven track record of use in clinical settings, where it has provided consistent bone regeneration results. It is also stable and does not absorb quickly, which allows it to maintain volume and structure for a long time, which is necessary to support implants in the case of extensive bone defects [25,26,27,28]. In this study, we used two types of bone substitute materials: Bio-Oss and Bio-Gide, which were supplied by Geistlich from Switzerland. We used 1 g of Bio-Oss and a 25 × 25 mm Bio-Gide membrane cut into a shape to adequately cover the socket entrance using a socket preservation technique (Figure 3B).

Guided bone regeneration, utilizing xenographic material and a collagen barrier membrane, was employed to treat bone defects in patients after implant removal who participated in a study. This method not only enabled the reconstruction of the bone defect, but also prepared the site for the stable placement of a new implant, which is crucial for long-term success in dental implantology.

Guided bone regeneration is an advanced technique used in dental implantology to restore bone defects, particularly after the removal of an implant. This technique utilizes xenographic material and a collagen barrier membrane, enabling effective bone regeneration and preparing the site for a new implant.

After implant removal, the defect site is carefully cleaned to remove any tissue remnants and ensure a clean surface for bone regeneration. Xenographic material, most commonly deproteinized bovine bone mineral (DBBM), is then applied to the defect site. DBBM acts as a scaffold for new bone, providing volumetric stability and supporting the osteogenesis process due to its low resorption rate.

The bone graft material is then covered with a collagen barrier membrane. The collagen membrane prevents soft tissue from infiltrating the regeneration area, creating an optimal environment for bone cells to effectively fill the defect. Due to its resorbable properties, the collagen membrane does not require a second surgery for removal, reducing the risk of complications and improving patient comfort.

The wound is closed tension-free with sutures to prevent dehiscence and promote proper tissue healing. The entire regeneration process is monitored to ensure proper bone regrowth and that the site is adequately prepared for the placement of a new implant [29].

### 2.3. Material Implementation Procedure

After the implant removal procedure, appropriate bone replacement materials were immediately placed in the sockets of patients in the Small-Particle Dentin and Bio-Oss Groups. The eye sockets of the Control Group were allowed to heal naturally. All surgical procedures were standardized to ensure consistency across procedures.

Prior to the use of bone substitute materials, all extraction sockets were thoroughly cleaned to remove any inflammatory tissue, preparing a clean substrate for the grafting procedure. Each material was used according to a standard protocol to ensure consistent treatment for all participants. Bio-Oss was thickened in the extraction sockets to ensure thorough filling, while the fine-particle dentin has been carefully molded to fit the contours of each socket, facilitating optimal adaptation and regenerative potential. Regular clinical examinations were performed to assess the healing and integration of the materials with the surrounding bony structures.

To assess the initial condition of the post-extraction funnels, baseline radiographs were taken. Follow-up radiographs were taken at specified intervals to monitor the progression of bone attenuation and the degree of socket filling.

### 2.4. Assessment of Radiographs and Assessment of Bone Attenuation

To determine the region of interest (ROI) in the volumetric analysis of bone tissue using manual segmentation, the methodology involved several key steps. CBCT images were processed using the OnDemand3D App, an application specifically designed for such analyses. The OnDemand3D App, version 1.0.11.1007, is produced by Cybermed. The polygon selection tool in OnDemand3D was utilized to manually outline the region of bone tissue on the selected slices. Specifically, square areas of 30 × 30 pixels were delineated to achieve precise segmentation of the ROI. This tool allowed for the measurement of minimum, maximum, average, and standard deviation density values within the specified region. To draw the region of interest, the tool options provided choices of [Rectangle], [Circle], or [Polyline], with the square (30 × 30 pixels) selection being used in this analysis.

For volume measurement, the ROI measurement tool in OnDemand3D was used to calculate the volume of bone tissue based on the outlined slices. Accurate slices were defined from a specified starting point to the endpoint within the analyzed bone, using appropriate reference points such as characteristic anatomical landmarks [30]. Bone attenuation values obtained from baseline scans were compared with the results of follow-up examinations after 8 weeks (for the Small-Particle Dentin Group) and 12 weeks (for the Bio-Oss Group and Control Group).

The study conducted by Martins L. A. C. et al. convincingly demonstrates the utility of CBCT in accurately assessing tissue density using Hounsfield units (HUs). The consistent and reliable HU measurements highlight CBCT’s ability to provide detailed and reproducible evaluations of bone density, which is crucial for successful dental implant planning. Furthermore, the strong correlation between CBCT grey values and tissue density underscores its potential as a reliable diagnostic tool, advocating for its broader clinical adoption to enhance implant placement outcomes [31].

**Figure 4:**
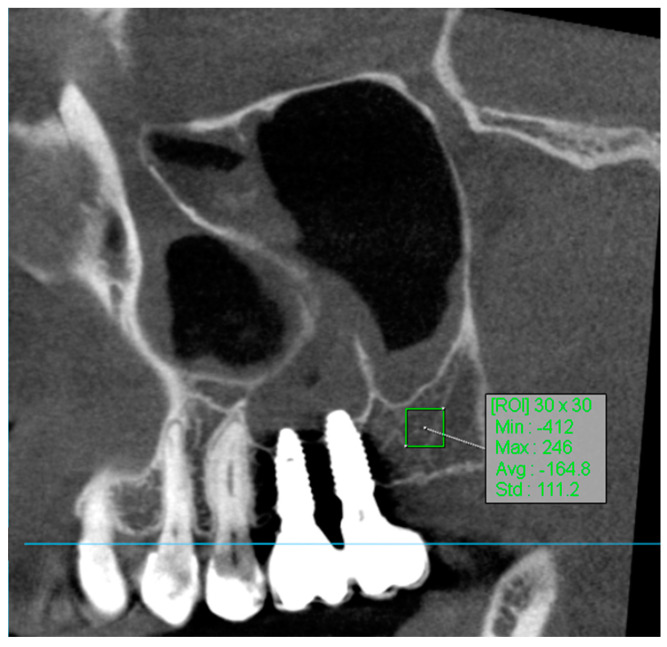
ROI measurement.

The use of regions of interest (ROI) in CBCT imaging is highly beneficial for accurately correlating gray values with Hounsfield Units (HUs) (Figure 4). This approach enhances diagnostic precision and treatment planning in dental radiology [32].

According to Kometabio’s protocol, the assessment of bone conditions at the application site should be performed 8 weeks after placement in the augmented area [33]. The strong osteoconductive properties of small-particle dentin allow for a faster evaluation compared to Bio-Oss material. Furthermore, when using small-particle dentin, a barrier membrane is not utilized, unlike in augmentations using Bio-Oss material and Bio-Gide membrane. By 12 weeks, healing is mainly characterized by ongoing filling of the intertrabecular spaces where maturation to lamellar bone begins, as described in the publication by Schwarz F et al. [34].

In order to assess the initial condition of the post-extraction funnels, baseline radiographs were taken. Follow-up radiographs were taken at specified intervals to monitor the progression of bone attenuation and the degree of socket filling. The primary radiological outcome measure was the change in bone attenuation over time compared with baseline measurements.

### 2.5. Statistical Analysis

The statistical analysis for this study was conducted using software StatSoft Statistica 13.1 PL package, for study power calculation. The sample size was determined based on preliminary data that showed the expected difference in bone regeneration results between the two materials. A power analysis was performed to determine the minimum number of participants required to detect a statistically significant difference with a power of 80% and a significance level of 0.05. It was calculated that a sample of 57 patients would be sufficient. The effect size for the stated study power analysis was Cohen’s d = 0.5. The parameter that the study power calculations were based on was the mean difference in bone attenuation. To ensure the reliability of our results, data were analyzed using Student’s *t*-test for dependent samples. This test is suitable for comparing the means in paired samples, such as the pre- and post-treatment bone attenuation measurements in this study. The choice of Student’s *t*-test resulted from its proven effectiveness in detecting the differences between paired groups assuming a normal distribution of data and homogeneity of variances. Before conducting Student’s *t*-test, normal distribution analysis of the data was performed using the Shapiro–Wilk test, which was used due to its high power in detecting deviations from normality in small samples. This analysis included a comparison of the mean changes in bone attenuation and clinical outcomes across study groups, using ANOVA for group comparisons and, where appropriate, post-hoc tests. Post-hoc tests used in the analysis included the Tukey test. To ensure the validity and reliability of the results, the threshold of statistical significance was set at *p* < 0.05 for all tests.

## 3. Results

The data obtained regarding the assessment of bone attenuation after the use of small-particle dentin and Bio-Oss in relation to patients from the Control Group are presented in Table 4.

The average bone attenuation is higher in the Bio-Oss Group (910.51) compared to the Small-Particle Dentin Group (779.62), though not statistically significant (*p* = 0.652). Both groups significantly outperform the Control Group (*p* < 0.001), indicating effective regeneration (Table 4).

No significant differences were found at adjacent sites, suggesting localized effects. Bio-Oss shows a higher mean difference in density (665.29 HU) versus small-particle dentin (419.88 HU), with a *p*-value of 0.072. Both groups significantly differ from the control (*p* < 0.001), confirming effective treatment.

The data indicate that both materials effectively increase bone attenuation, with Bio-Oss potentially being more effective. The effects are localized to treated areas.

In the next step, bone attenuation (in the tested place and in the vicinity) was compared in individual research groups after the use of the tested regenerative materials, as illustrated in Table 5.

The average bone attenuation for Bio-Oss in the study site (910.51 HU) is significantly higher than in the adjacent site (245.23 HU), with a *p*-value < 0.001, indicating a significant difference. Similarly, the average bone density for fine dentin in the study site (779.62 HU) is significantly higher than in the adjacent site (359.75 HU), with a *p*-value < 0.001 (Table 5).

In the Control Group, bone attenuation in the study site (206.04 HU) is lower than in the adjacent site (272.19 HU), but this difference is not statistically significant. Both Bio-Oss and Small-Particle Dentin Groups showed significant increases in bone attenuation at the test site compared to the adjacent site. The Control Group showed no significant difference.

We also decided to analyze the obtained research results in terms of bone attenuation assessment (in the tested place and in the vicinity) in individual research groups after the use of the tested regenerative materials, taking into account the anatomical location, which is presented in Table 6 and in detail in Table 7.

The analyses conducted show that statistically significant differences were observed in the research center between Small-Particle Dentin and Bio-Oss Groups and the Control Group in both the mandible and maxilla. No statistically significant differences were observed between both research groups. Additionally, a statistically significant difference in bone density was also observed for the Small-Particle Dentin and Bio-Oss Groups and the mandibular Control Group, and only for Bio-Oss and the maxillary Control Group.

Bone attenuation in the Bio-Oss Group was significantly higher at the studied site compared to the adjacent site for both mandible and maxilla (*p* < 0.001), indicating its effectiveness in increasing bone density (Table 7). Similarly, the Small-Particle Dentin Group shows significantly higher bone attenuation at the studied site for both the mandible (*p* < 0.001) and maxilla (*p* = 0.018), demonstrating its efficacy (Table 7). In the Control Group, there are no significant differences in bone attenuation between studied and adjacent sites for both mandible (*p* = 0.278) and maxilla (*p* = 0.391), indicating no significant increase in bone density without regenerative materials (Table 7).

Both Bio-Oss and Small-Particle Dentin significantly increase bone density at the treated sites in the mandible and maxilla, with Bio-Oss showing a highly significant increase. The Control Group shows no significant differences, highlighting the impact of the tested materials.

In the next stage, we decided to analyze the results of the obtained research in the context of differences occurring in individual genders. Detailed data are presented in Table 8 and Table 9.

For female patients, the mean bone density was 689.17 HU for the small-particle dentin, 920.28 HU for Bio-Oss, and 229.67 HU for the Control Group. Bio-Oss and small-article dentin significantly outperformed the control (*p* < 0.001), but not each other (*p* = 0.354).

For male patients, the mean bone density was 973.45 HU for small-particle dentin, 899.35 HU for Bio-Oss, and 111.54 HU for the control. Both Bio-Oss and small-particle dentin significantly outperformed the control (*p* < 0.05), but not each other (*p* = 1.000).

At adjacent sites, no significant differences in bone density were observed among the groups.

Both materials significantly increase bone density at treated sites, with Bio-Oss showing particularly strong effects. The differences at adjacent sites were not significant, indicating localized effectiveness.

For female patients in the Bio-Oss Group, bone density was significantly higher at the studied site (920.28 HU) than the adjacent site (232.70 HU, *p* < 0.001). For male patients, the studied site density was 899.35 HU, also higher than the adjacent site (259.55 HU, *p* < 0.001. In the Small-Particle Dentin Group, for females, the studied site density was 689.17 HU, with no significant difference at the adjacent site (330.37 HU, *p* = 0.504). For males, the studied site density was 973.45 HU, nearly significant compared to the adjacent site (422.70 HU, *p* = 0.063) (Table 9).

In the Control Group, for females, the studied site density was 229.67 HU, lower than the adjacent site (265.75 HU, *p* < 0.001). For males, the studied site density was 111.54 HU, lower than the adjacent site (297.94 HU, *p* = 0.006) (Table 9).

## 4. Discussion

**Figure 5:**
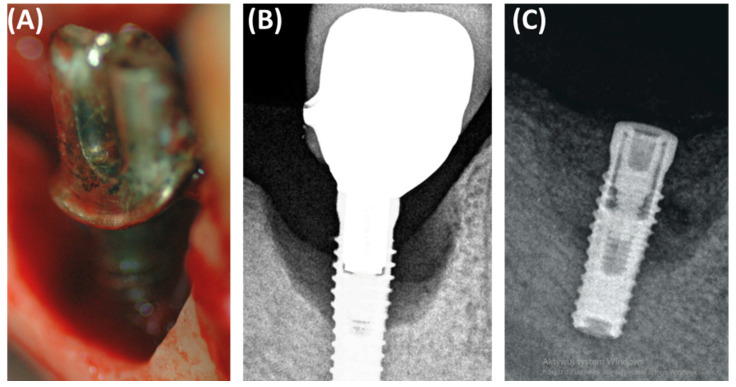
Images showing the defect resulting from periimplantitis: (**A**) clinical photo, (**B**) radiographic photo, (**C**) photo after defect restoration with bone substitute material.

In the context of peri-implantitis, effective treatment of bone defects is crucial for successful implant treatment (Figure 5). Small-particle dentin has proven to be a promising bone replacement material due to its biological compatibility and osteoconductive properties [33]. Sourced from human teeth after extraction, the fine-particle dentin is processed to remove any organic contaminants while retaining its natural, mineral-rich structure [35]. This makes the material biocompatible and supports the growth of new bone, acting as an osteoconductive scaffold that facilitates the adhesion and proliferation of new bone cells, making it ideal for socket preservation and the repair of bone defects caused by peri-implantitis [36]. This biological material has several advantages over synthetic bone substitutes. The high biocompatibility of fine dentin reduces the risk of immune rejection and adverse reactions, offering a cost-effective solution by using autologous materials and reducing the environmental impact of medical waste. Studies have demonstrated the effectiveness of fine-particle dentin in supporting bone regeneration, emphasizing its role in increasing the dimensions of the alveolar ridge necessary in implantology and demonstrating better healing times and better results compared to other graft materials [37].

Bio-Oss, a natural bone substitute of bovine origin, is widely used in dentistry and orthopedics due to its unique osteoconductive properties and structural similarity to human bone. The Bio-Oss production process includes rigorous cleaning and sterilization to remove all organic ingredients, ensuring its safety and high biocompatibility. This material mimics the mineral composition and porosity of human bone, which facilitates integration into the bone structure and acts as an effective scaffold for new bone growth [38]. Bio-Oss is mainly used to fill bone defects and augment bone in areas affected by peri-implantitis, significantly improving the structural stability of dental implants. Its stability and resistance to rapid resorption make it essential for supporting implants in the case of extensive bone defects. Empirical studies confirm the use of Bio-Oss in the treatment of peri-implantitis, showing that it not only supports but also intensifies bone regenerative processes, contributing to improved healing results and integration with the host bone tissue [39,40].

In a study conducted by our team, the effectiveness of small-particle dentin and Bio-Oss was assessed in the context of bone regeneration in the case of defects caused by peri-implantitis. The results indicate that both materials significantly improved bone density compared to the control group that did not receive any bone replacement material. Fine-molecular dentin facilitated rapid initial bone regeneration, while Bio-Oss provided durable and long-term support [36,41,42,43]. The use of diode lasers in the treatment of peri-implantitis effectively reduces the microbial load on titanium implants, increasing the overall effectiveness of treatment by creating a cleaner environment conducive to bone regeneration [44,45,46].

These findings align with previous research that has shown that bone replacement materials significantly improve bone regeneration outcomes. Both immediate and long-term improvements have been observed when choosing between fine-particle dentin and Bio-Oss based on specific clinical scenarios, patient needs, and desired treatment outcomes [46,47]. Further research into innovative approaches to flap design, surface decontamination, and use of bone graft materials is necessary to optimize peri-implantitis treatment protocols and improve long-term outcomes [14,48,49,50,51,52].

Although the presented research results are promising, it is important to mention the limitations of the study that our research team encountered when conducting these analyses. First of all, the study included a relatively small number of patients (57), which may limit the generalization of the results obtained to a wider population. Larger samples are necessary to confirm the results and ensure their representativeness. Although the results suggest the effectiveness of fine-particle dentin and Bio-Oss in bone regeneration, the follow-up period (12 weeks) may be too short to evaluate the long-term effects of these materials. We believe that longer studies are necessary to better understand the durability and stability of regenerative outcomes. The study may not have taken into account sufficient demographic and clinical diversity among patients. Factors such as age, gender, general health, and lifestyle (e.g., smoking, drinking habits) may significantly influence the process of bone healing and regeneration, which should be taken into account in future studies. The chosen methodology should also be stated as a limitation, as it may have affected the outcomes and interpretations of the results. Specifically, the use of Hounsfield Units (HU) is not the most suitable approach for bone assessments, which presents a limitation in the chosen methodology. The type and severity of bone defects may vary, which affects treatment results. The study may not have included different classifications of bone defects (e.g., subosseous, horizontal, complex defects), which may limit the ability to compare results in different clinical scenarios. Although the study provides valuable data on the effectiveness of small-particle dentin and Bio-Oss in bone regeneration after peri-implantitis, addressing the above limitations in future studies is crucial to confirm and extend the results obtained.

## 5. Conclusions

This study demonstrated the significant benefits of using bone replacement materials in the treatment of bone defects caused by peri-implantitis. Both fine-particle dentin and Bio-Oss are effective in supporting bone regeneration, and each has unique benefits.

Fine-particle dentin is characterized by immediate bone regeneration, showing rapid healing and high biocompatibility within eight weeks. It is ideal for urgent clinical applications, preserving alveolar ridge dimensions for subsequent dental implants.

Bio-Oss provide a solid foundation for new bone growth with a steady increase in bone density over the long term. It is suitable for long-term stability in cases of extensive bone loss, ensuring reliable stability of the graft site.

Both materials are characterized by high integration with native bone, with fine-particle dentin characterized by faster healing and slightly greater effectiveness. The use of diode lasers increases the effectiveness of these materials by reducing the microbial load.

Selecting the appropriate bone replacement material based on clinical scenarios and patient needs is crucial. Fine-particle dentin is recommended for immediate regeneration, while Bio-Oss is preferred for long-term stability. Future research should examine these materials across a variety of bone defects and patient demographics to optimize treatment protocols for peri-implantitis.

## Figures and Tables

**Table 1:** Characteristics of the selected parameters of the patients recruited for the study divided into individual research groups.

Parameter		Small-Particle Dentin Group (Group 1) (HU)	Bio-Oss Group (Group 2) (HU)	Control Group (Group 3) (HU)	*p*-Value		
		Mean ± SD Median (Range)	Mean ± SD Median (Range)	Mean ± SD Median (Range)	1 vs. 2	1 vs. 3	2 vs. 3
Age		27.91 ± 11.1 24 (18–58)	40.27 ± 12.79 40 (19–60)	29.15 ± 8.52 27 (16–58)	0.002 *	0.495	0.096
Bone attenuation		779.62 ± 325.92 777.63 (185.16–1467.64)	910.51 ± 155.03 936.37 (677.01–1140.91)	206.04 ± 174.21 156.52 (14.73–672.74)	0.652	<0.001 *	<0.001 *
Sex	Female	15 (68.18%)	8 (53.33%)	16 (80.00%)	1.000	1.000	0.278
	Male	7 (31.82%)	7 (46.67%)	4 (20.00%)			
Anatomical area	Maxilla	10 (45.45%)	10 (66.67%)	9 (45.00%)	0.611	1.000	0.609
	Mandible	12 (54.55%)	5 (33.33%)	11 (55.00%)			

* statistically significant results marked.

**Table 2:** Characteristics of the selected parameters of the patients recruited for the study divided into the anatomical area of changes in individual groups of patients.

Parameter			Small-Particle Dentin Group (Group 1) (HU)		Bio-Oss Group (Group 2) (HU)		Control Group (Group 3) (HU)		*p*-Value		
			Mean ± SD	Median (Range)	Mean ± SD	Median (Range)	Mean ± SD	Median (Range)	1 vs. 2	1 vs. 3	2 vs. 3
Mandible	Age		26.42 ± 10.18	24 (18–58)	52.60 ± 8.23	52 (40–60)	29.18 ± 10.79	26 (16–58)	0.003 *	0.634	0.061
	Bone attenuation		736.07 ± 205.15	728.45 (472.41–1082.07)	865.70 ± 173.86	758.83 (736.45–1140.91)	235.05 ± 168.81	203.46 (42.86–672.74)	1.000	0.001 *	0.001 *
	Sex	F	9 (75.00%)		3 (60.00%)		9 (81.82%)		1.000	1.000	1.000
		M	3 (25.00%)		2 (40.00%)		2 (18.18%)				
Maxilla	Age		29.70 ± 12.42	24.5 (18–50)	34.10 ± 9.88	33.5 (19–52)	29.11 ± 5.18	28 (24–41)	0.567	1.000	1.000
	Bone attenuation		831.89 ± 436.86	811.96 (185.16–1467.64)	932.92 ± 149.26	978.61 (677.01–1108.53)	170.59 ± 184.09	74.54 (14.73–607.06)	1.000	0.003 *	0.001 *
	Sex	F	6 (60.00%)		5 (50.00%)		7 (77.78%)		1.000	1.000	0.630
		M	4 (40.00%)		5 (50.00%)		2 (22.22%)				

Abbreviations: F—female; M—male; * statistically significant results marked.

**Table 3:** Characteristics of the selected parameters of the patients recruited for the study by gender in individual patient groups.

Parameter			Small-Particle Dentin Group (Group 1) (HU)		Bio-Oss Group (Group 2) (HU)		Control Group (Group 3) (HU)		*p*-Value		
			Mean ± SD	Median (Range)	Mean ± SD	Median (Range)	Mean ± SD	Median (Range)	1 vs. 2	1 vs. 3	2 vs. 3
Female	Age		29.60 ± 12.11	24 (19–58)	38.00 ± 15.51	37.5 (19–60)	27.63 ± 8.74	26 (16–58)	0.226	1.000	0.710
	Bone attenuation		689.17 ± 328.94	588.69 (185.16–1467.64)	920.28 ± 132.96	915.35 (755.95–1140.91)	229.67 ± 185.89	219.89 (42.86–672.74)	0.354	0.002 *	<0.001 *
	Anatomical area	Maxilla	6 (40.00%)		5 (62.50%)		7 (43.75%)		0.911	1.000	1.000
		Mandible	9 (60.00%)		3 (37.50%)		9 (56.25%)				
Male	Age		24.29 ± 8.16	25 (18–41)	42.86 ± 9.30	42 (31–52)	35.25 ± 3.95	34 (32–41)	0.008 *	0.235	1.000
	Bone attenuation		973.45 ± 234.58	914.09 (696.44–1271.57)	899.35 ± 187.56	936.37 (677.01–1108.53)	111.54 ± 67.52	130.58 (14.73–170.27)	1.000	0.010 *	0.042 *
	Anatomical area	Maxilla	4 (57.14%)		5 (71.43%)		2 (50.00%)		1.000	1.000	1.000
		Mandible	3 (42.86%)		2 (28.57%)		2 (50.00%)				

* statistically significant results marked.

**Table 4:** Assessment of bone density (in the tested site and the adjacent site) in individual study groups after the use of the tested regenerative materials.

Bone Attenuation	Small-Particle Dentin Group (Group 1) (HU)		Bio-Oss Group (Group 2) (HU)		Control Group (Group 3) (HU)		*p*-Value		
	Mean ± SD	Median (Range)	Mean ± SD	Median (Range)	Mean ± SD	Median (Range)	1 vs. 2	1 vs. 3	2 vs. 3
At the studied site	779.62 ± 325.92	777.63 (185.16–1467.64)	910.51 ± 155.03	936.37 (677.01–1140.91)	206.04 ± 174.21	156.52 (14.73–672.74)	0.652	<0.001 *	<0.001 *
In the adjacent site	359.75 ± 195.10	378.00 (84.95–807.58)	245.23 ± 155.29	207.29 (23.42–597.78)	272.19 ± 153.06	289.30 (43.15–562.89)	0.183	0.456	1.000
Difference in density	419.88 ± 317.60	346.99 (−116.32–1120.95)	665.29 ± 142.31	687.37 (413.81–884.77)	−66.14 ± 203.62	−43.45 (−490.11–288.83)	0.072	<0.001 *	<0.001 *

* statistically significant results marked.

**Table 5:** Comparative analysis of bone attenuation (in the tested site and the adjacent site) in individual study groups after the use of the tested regenerative materials.

Patient Group	At the Studied Site (HU)		In the Adjacent Site (HU)		*p*-Value
	Mean ± SD	Median (Range)	Mean ± SD	Median (Range)	
Bio-Oss Group	910.51 ± 155.03	936.37 (677.01–1140.91)	245.23 ± 155.29	207.29 (23.42–597.78)	<0.001 *
Small-Particle Dentin Group	779.62 ± 325.92	777.63 (185.16–1467.64)	359.75 ± 195.10	378.00 (84.95–807.58)	<0.001 *
Control group	206.04 ± 174.21	156.52 (14.73–672.74)	272.19 ± 153.06	289.30 (43.15–562.89)	0.163

* statistically significant results marked.

**Table 6:** Assessment of bone density (in the tested site and the adjacent site) in individual study groups after the use of the tested regenerative materials, taking into account the anatomical location.

Bone Attenuation		Small-Particle Dentin Group (Group 1) (HU)		Bio-Oss Group (Group 2) (HU)		Control Group (Group 3) (HU)		*p*-Value		
		Mean ± SD	Median (Range)	Mean ± SD	Median (Range)	Mean ± SD	Median (Range)	1 vs. 2	1 vs. 3	2 vs. 3
Mandible	At the studied site	736.07 ± 205.15	728.45 (472.41–1082.07)	865.70 ± 173.86	758.83 (736.45–1140.91)	235.05 ± 168.81	203.46 (42.86–672.74)	1.000	0.001 *	0.001 *
	In the adjacent site	319.51 ± 148.55	337.21 (107.53–562.89)	245.42 ± 117.11	186.36 (184.40–453.54)	293.36 ± 147.89	280.15 (98.96–562.89)	1.000	1.000	1.000
	Difference in density	416.56 ± 132.12	364.88 (265.07–687.81)	620.29 ± 77.37	572.47 (552.05–719.95)	−58.32 ± 168.64	−43.06 (−319.72–207.01)	0.317	0.002	<0.001 *
Maxilla	At the studied site	831.89 ± 436.86	811.96 (185.16–1467.64)	932.92 ± 149.26	978.61 (677.01–1108.53)	170.59 ± 184.09	74.54 (14.73–607.06)	1.000	0.003 *	0.001 *
	In the adjacent site	408.03 ± 238.95	382.28 (84.95–807.58)	245.13 ± 177.25	217.26 (23.42–597.78)	246.30 ± 164.11	308.64 (43.15–562.89)	0.278	0.329	1.000
	Difference in density	423.86 ± 462.60	192.01 (−116.32–1120.95)	687.79 ± 164.79	752.60 (413.81–884.77)	−75.70 ± 250.46	−52.69 (−490.11–288.83)	0.468	0.055	0.001 *

* statistically significant results marked.

**Table 7:** Comparative analysis of bone attenuation (in the tested site and the adjacent site) in individual study groups after the use of the tested regenerative materials, taking into account the anatomical location.

Patients Group	Anatomical Location	At the Studied Site (HU)		In the Adjacent Site (HU)		*p*-Value
		Mean ± SD	Median (Range)	Mean ± SD	Median (Range)	
Bio-Oss Group	Mandible	865.70 ± 173.86	758.83 (736.45–1140.91)	245.42 ± 117.11	186.36 (184.40–453.54)	<0.001 *
	Maxilla	932.92 ± 149.26	978.61 (677.01–1108.53)	245.13 ± 177.25	217.26 (23.42–597.78)	<0.001 *
Small-Particle Dentin Group	Mandible	736.07 ± 205.15	728.45 (472.41–1082.07)	319.51 ± 148.55	337.21 (107.53–562.89)	<0.001 *
	Maxilla	831.89 ± 436.86	811.96 (185.16–1467.64)	408.03 ± 238.95	382.28 (84.95–807.58)	0.018 *
Control group	Mandible	235.05 ± 168.81	203.46 (42.86–672.74)	293.36 ± 147.89	280.15 (98.96–562.89)	0.278
	Maxilla	170.59 ± 184.09	74.54 (14.73–607.06)	246.30 ± 164.11	308.64 (43.15–562.89)	0.391

* statistically significant results marked.

**Table 8:** Assessment of bone attenuation (in the tested site and the adjacent site) in individual study groups after the use of the tested regenerative materials, taking into account sex.

Bone Attenuation		Small-Particle Dentin Group (Group 1) (HU)		Bio-Oss Group (Group 2) (HU)		Control Group (Group 3) (HU)		*p*-Value		
		Mean ± SD	Median (Range)	Mean ± SD	Median (Range)	Mean ± SD	Median (Range)	1 vs. 2	1 vs. 3	2 vs. 3
Female	At the studied site	689.17 ± 328.94	588.69 (185.16–1467.64)	920.28 ± 132.96	915.35 (755.95–1140.91)	229.67 ± 185.89	219.89 (42.86–672.74)	0.354	0.002 *	<0.001 *
	In the adjacent site	330.37 ± 175.92	358.11 (84.95–674.57)	232.70 ± 163.65	187.24 (23.42–498.26)	265.75 ± 162.12	271.19 (43.15–562.89)	0.480	0.884	1.000
	Difference in density	322.28 (−116.32–1109.53)	358.80 ± 293.47	687.59 ± 123.97	704.54 (511.65–834.25)	−36.08 ± 210.62	−14.04 (−490.11–288.83)	0.104	0.004 *	<0.001 *
Male	At the studied site	973.45 ± 234.58	914.09 (696.44–1271.57)	899.35 ± 187.56	936.37 (677.01–1108.53)	111.54 ± 67.52	130.58 (14.73–170.27)	1.000	0.010 *	0.042 *
	In the adjacent site	422.70 ± 232.87	431.37 (150.62–807.58)	259.55 ± 156.76	216.42 (110.16–597.78)	297.94 ± 125.89	317.21 (127.16–430.18)	0.440	1.000	1.000
	Difference in density	550.75 ± 350.40	438.87 (228.52–1120.95)	639.80 ± 167.05	602.04 (413.81–884.77)	−186.40 ± 129.09	−223.52 (−293.91–−4.65)	1.000	0.053	0.008 *

* statistically significant results marked.

**Table 9:** Comparative analysis of bone attenuation (in the tested site and the adjacent site) in individual study groups after the use of the tested regenerative materials, taking into account sex.

Patient Group	Anatomical Location	At the Studied Site (HU)		In the Adjacent Site (HU)		*p*-Value
		Mean ± SD	Median (Range)	Mean ± SD	Median (Range)	
Bio-Oss Group	Female	920.28 ± 132.96	915.35 (755.95–1140.91)	232.70 ± 163.65	187.24 (23.42–498.26)	<0.001 *
	Male	899.35 ± 187.56	936.37 (677.01–1108.53)	259.55 ± 156.76	216.42 (110.16–597.78)	<0.001 *
Small-Particle Dentin Group	Female	689.17 ± 328.94	588.69 (185.16–1467.64)	330.37 ± 175.92	358.11 (84.95–674.57)	0.504
	Male	973.45 ± 234.58	914.09 (696.44–1271.57)	422.70 ± 232.87	431.37 (150.62–807.58)	0.063
Control group	Female	229.67 ± 185.89	219.89 (42.86–672.74)	265.75 ± 162.12	271.19 (43.15–562.89)	<0.001 *
	Male	111.54 ± 67.52	130.58 (14.73–170.27)	297.94 ± 125.89	317.21 (127.16–430.18)	0.006 *

* statistically significant results marked.

## Data Availability

Research data resulting from the implementation of this study are available upon written request from the corresponding author.
